# Can metamorphosis survival during larval development in spiny lobster *Sagmariasus verreauxi* be improved through quantitative genetic inheritance?

**DOI:** 10.1186/s12863-018-0621-z

**Published:** 2018-05-04

**Authors:** Nguyen H. Nguyen, Quinn P. Fitzgibbon, Jane Quinn, Greg Smith, Stephen Battaglene, Wayne Knibb

**Affiliations:** 1 GeneCology Research Center, The University of the Sunshine Coast, Maroochydore DC, QLD 4558 Australia; 2 Fisheries and Aquaculture, Institute of Marine and Antarctic Studies, University of Tasmania, Private Bag 49, Hobart, TAS 7001 Australia

**Keywords:** Genetic variation, Genetic correlations, Selective breeding and lobsters

## Abstract

**Background:**

One of the major impediments to spiny lobster aquaculture is the high cost of hatchery production due to the long and complex larval cycle and poor survival during the many moult stages, especially at metamorphosis. We examined if the key trait of larval survival can be improved through selection by determining if genetic variance exists for this trait. Specifically, we report, for the first time, genetic parameters (heritability and correlations) for early survival rates recorded at five larval phases; early-phyllosoma stages (instars 1–6; S1), mid-phyllosoma stages (instars; 7–12; S2), late-phyllosoma stages (instars 13–17; S3), metamorphosis (S4) and puerulus stage (S5) in hatchery-reared spiny lobster *Sagmariasus verreauxi*.

**Results:**

The data were collected from a total of 235,060 larvae produced from 18 sires and 30 dams over nine years (2006 to 2014). Parentage of the offspring and full-sib families was verified using ten microsatellite markers. Analysis of variance components showed that the estimates of heritability for all the five phases of larval survival obtained from linear mixed model were generally similar to those obtained from threshold logistic generalised models (0.03–0.47 vs. 0.01–0.50). The heritability estimates for survival traits recorded in the early larval phases (S1 and S2) were higher than those estimated in later phases (S3, S4 and S5). The existence of the additive genetic component in larval survival traits indicate that they could be improved through selection. Both phenotypic and genetic correlations among the five survival measures studied were moderate to high and positive. The genetic associations between successive rearing periods were stronger than those that are further apart.

**Conclusions:**

Our estimates of heritability and genetic correlations reported here in a spiny lobster species indicate that improvement in the early survival especially during metamorphosis can be achieved through genetic selection in this highly economic value species.

**Electronic supplementary material:**

The online version of this article (10.1186/s12863-018-0621-z) contains supplementary material, which is available to authorized users.

## Background

Spiny lobsters are one of the world’s most valuable seafood commodities, however, closed life cycle commercial aquaculture has not been possible due to difficulties in culturing larvae during the long and complicated development phases [1]. Larval development of spiny lobsters is dominated by the phyllosoma phase where larvae moult through numerous instars before metamorphosing into puerulus post-larva which in the wild is the nektonic link between planktonic phyllosoma and benthic juvenile development [2]. Advancements in the hatchery production of the spiny lobster, *Sagmariasus verreauxi*, at the University of Tasmania has opened new opportunities to promote commercial aquaculture and also undertake genetic improvement of the species [3, 4]. As a first step of any genetic improvement program, genetic properties and quantitative genetic inheritance of economically important or desired traits are needed to assist in the choice of selection criteria, development of breeding objectives, genetic evaluation systems as well as selection and mate allocation methods [5, 6]. To our knowledge, there are no published data on the genetic basis of quantitative economic traits in any spiny lobster species. In spiny lobsters there is limited knowledge of the genetic parameters (heritability and correlations) governing complex traits. Studies in other crustaceans species show that there is abundant additive genetic variation for morphometric characters related to growth. Heritability estimates ranging from 0.10 to 0.60 are present in marine shrimps *Penaeus monodon* [7], *Liptopenaeus vannamei* [8], *Fenneropenaeus merguiensis* [9, 10] and the giant freshwater prawn *Macrobrachium rosenbergii* [11, 12]. The moderate to high heritability for growth traits are in accordance with the positive response to selection, averaging 10% per generation or per year [13, 14].

In addition to growth characteristics, survival especially during metamorphosis, is another trait of commercial importance because it determines yield/or productivity and thereby affects profit and economic return of hatchery operators, growers and production sectors. Survival rate during grow-out is heritable in freshwater fishes [15–17], as is survival during the early phase of rearing for rainbow trout *Oncorhynchus mykiss*, tilapia *Oreochromis niloticus* and white leg shrimp *L. vannamei* [18, 19]. Low larval survival remains a key bottleneck for commercial closed cycle production for spiny lobsters. *Sagmariasus verreauxi* moult through 17 phyllosoma instars which takes from 6 and 8 months in hatchery production before they metamorphose to puerulus; a 21 days period, before moulting to juvenile [20, 21]. Relatively high mortality rates (> 15%) at each moult can result in overall low survival (≈5%) due to the large number of instar stages [22]. In addition, there has been a final bottle-neck to hatchery production at metamorphosis where mortality can be > 50% [23]. To our knowledge there are currently no reports on heritable genetic variation in larval survival characters of spiny lobsters and it is unknown if this trait can be improved with selection. The only estimate of heritability for any lobster trait was reported 32 years ago for aggressive behaviour in clawed lobsters, *Homarus americanus* [24] based on conventional analysis of variance, not mixed model methodologies.

To gain new knowledge in quantitative genetic characteristics for this emerging aquaculture species, we computed genetic parameter estimates (heritability and correlations) for measures of five larval survival phases, including metamorphosis, of *S. verreauxi*. The study was conducted using data from over nine successive years of hatchery production from 2006 to 2014.

## Methods

### Experimental location and animals

Data were collected at the Institute of Marine and Antarctic Studies (IMAS) in Hobart, Tasmania, Australia. Foundation stocks of *S. verreauxi* were collected from the wild as early instar juveniles off the East coast of Tasmania and held until reproductively active. They were kept in 4000 l fibreglass tanks under ambient photoperiod and water temperature (11 °C–19 °C), 33–35 ‰ salinity, pH approximately 8.1, and 90–100% oxygen saturation at IMAS [3]. In addition, the 2012 cohorts were bred from F1 generation broodstock. All broodstock were fed a combination of fresh whole blue mussels (*Mytilus galloprovincialis*) and commercial shrimp pellet twice a week.

### Family production and rearing

The average body weight of female and male *S. verreauxi* prior to breeding were 2.5 and 3.5 kg, respectively. A general description of breeding, larval rearing and growout is reported in earlier studies [3]. In brief, mating was practised in 4000 l tanks, with a ratio of 4 males to 8 females. Rearing of phyllosoma from each family to final instar (instar 17) was conducted separately in 200 l cylindrical fiberglass vessels at 21–23 °C. Larval rearing vessels were initially stocked with newly-hatched phyllosoma at a density of 12.5 l^− 1^, reduced to 7.5 l^− 1^ during mid-stage and 1 l^− 1^ during the late phyllosoma stages. Parentage of the offspring and full-sib families was verified using newly developed DNA markers (section 2.3 below). During phyllosoma rearing, larvae were fed with juvenile brine shrimp (*Artemia* sp.), blue mussel gonad and/or a ‘commercial in confidence’ manufactured diet. Individual final instar phyllosoma (instar 17) were transferred from mass culture tanks and were held in isolation for metamorphosis before pueruli were transferred to mass holding tanks until they moulted to the juvenile phase.

### Data recording

The survival data for hatchery reared *S. verreauxi* were determined at five larval rearing phases, early-phyllosoma stages (instars 1–6, culture days ≈ 0–36; (S1), mid-phyllosoma stages (instars; 7–12, culture days ≈37–150; S2), late-phyllosoma stages (instars 13–17, culture days ≈151–220; S3), metamorphosis (culture days ≈220; S4) and puerulus stage (culture days ≈221–242; S5). Phyllosoma survival measures were determined at routine density reductions at the mid- and late-phyllosoma stages and during isolation of final instar phyllosoma before metamorphosis. Early and mid-phyllosoma survival was determined by volumetric counts of larval numbers whereby five subsamples of larvae were counted and the average used to determine the total number of animals being cultured. Late stage phyllosoma were counted individually when removed from mass rearing vessels for metamorphosis. Metamorphosis survival was defined as survival from final phyllosoma instar (instar 17) to 2 days’ post metamorphosis. For each family cohort a minimum of three culture vessels were monitored for survival and the mean survival between the vessels indicated cohort survival.

Body weight of all individually tagged female broodstock was recorded using a digital scale following the annual moult cycle and before egg extrusion. The survival data at the five time points (S1 to S5) during the larval rearing was treated as binary characteristics and analysed using both linear and threshold mixed models (section 2.5).

### Development of new microsatellite markers, genotyping and parentage verification

A set of eleven microsatellite markers with consistent PCR amplification, clear allelic variation, and clarity of electrophoretic signatures were used to construct the pedigree. These highly polymorphic markers were developed using information from the transcriptome sequences of two *S. verreauxi* individuals [25]. Once validated in three multiplex PCR pools, each set containing 3 or 4 microsatellite primer pairs were amplified using Qiagen Multiplex PCR Plus Kits (Qiagen, Germany) in Eppendorf Mastercycler (Hamburg, Germany) with cycling conditions as follows: initial denaturation at 95 °C for 5 min, followed by 35 cycles of 94 °C for 30 s, 57 °C for 90 s, and 72 °C for 30 s; with a final extension at 68 °C for 10 min. Capillary separation of PCR products were conducted on an AB 3500 Genetic Analyser (Applied Biosystems). Subsequent fragment sizing analyses used GENEMARKER v1.95 software (SoftGenetics; State College, USA), with an internal control sample run with each batch (GS-600 LIZ; Applied Biosystems). Population genetic analysis was applied to check for evidence of large allele dropout, extreme stuttering and null alleles, as well to test for HWE at each locus and genotypic linkage equilibrium among pairs of loci, numbers of alleles, heterozygosities of each locus and polymorphic information content (*PIC*) (Additional file 1 Table S1). Parentage assignment was completed using COLONY software [26] with confidence scores above 95%.

### Statistical analysis

Heritability for survival traits were analysed using linear mixed models (LMM) and threshold logistic generalised mixed models (TLGM) in ASReml 3.0 [27]. For the LMM, the model included the additive genetic effect of individual lobster and the common full-sib effect as well the fixed effects of spawning year and live feeds (Model 1). The general form of the LMM model is written in mathematical notations as follows:

Model 1 $$ {y}_{ijklmn}=\mu +{F}_i+{Y}_j+{FW}_k+{a}_l+{c}_m+{e}_{ijklmn} $$

where *y*_*ijklmn*_ is the observation for survival traits studied of the individual *n*^*th*^; *μ* is the overall mean; *F* and *Y* are the fixed effects of live feeds and spawning years, respectively. *FW*_*k*_ is linear co-variable of average family weight of females before spawning; *a*_*l*_ is the additive genetic effect of individual lobsters; *c*_*m*_ is the common full-sib effect; and *e*_*ijklmn*_ is the random residual effect associated with individual *ijklmn*.

Heritability for survival traits were estimated from a single trait model as ${h}^2=\frac{{\widehat{\sigma}}_a^2}{{\widehat{\sigma}}_a^2+{\widehat{\sigma}}_c^2+{\widehat{\sigma}}_e^2}$ where ${\sigma}_a^2$ is the additive genetic variance component, and ${\sigma}_c^2$ is the common full-sib vairance and ${\sigma}_e^2$ is the residual/evnrionmetal variance. Genetic and phenotypic correlations between survival traits (S1, S2, S3, S4 and S5) were estimated from a series of bivariate analyses, using the same statistical model as described in Model 1. The phenotypic and genetic correlations were calculated as the covariance divided by the product of the standard deviations of traits: $r=\frac{\sigma_{12}}{\sqrt{\sigma_1^2}\sqrt{\sigma_2^2}}$ where *σ*_12_ was the estimated additive genetic or phenotypic covariance between the two traits, and ${\sigma}_1^2$ and ${\sigma}_2^2$ are the additive genetic or phenotypic variances of traits 1 and 2, respectively.

As survival traits were recorded as binary characters (coded as 1 for lobsters that were present at instar 6, 12, 17, and puerulus and 0 for those that were absent), the threshold logistic generalised mixed models (TLGM) were also used because they may have better explained genetic variation in survival characters. The threshold logistic generalised model (Model 2) assumed that the data followed a binomial distribution with a logit link functions $\left(\widehat{\mathrm{p}}={\mathrm{e}}^{\mathrm{x}}/\left(1+{\mathrm{e}}^{\mathrm{x}}\right)\right)$ where *p* is the survival probability of individual lobster recorded at five different nursing periods and *x* is a linear predictor. The class variable effects were the same as described in Eq. 1. The random effects included both the additive genetic effect of individual lobster and common full-sib effects (Model 2).

Model 2 $$ \log \left(\frac{p_{ijklmn}}{1-{p}_{ijklmn}}\right)=\mu +{F}_i+{Y}_j+{FW}_k+{a}_l+{c}_m+{e}_{ijklmn} $$

Under model 2, the heritability was calculated as

$$ {h}^2=\frac{\sigma_A^2}{\sigma_A^2+{\sigma}_c^2+{\sigma}_e^2\frac{\pi^2}{3}} $$

For binomial observations, estimates of *h*^*2*^ on the observed scale (0/1) were transformed to the liability scales (logit and probit) using the formula of Robertson and Lerner [28] as follows:

$$ {h}_L^2=\frac{h_o^2p\left(1-p\right)}{z^2} $$

where ${h}_o^2$ is the heritability on the observed (0/1) scale, ${h}_L^2$ is the estimated heritability on the liability (logit or probit) scale, *p* is a proportion of a given survival rate in the data, and *z* is the height of the ordinate of normal distribution corresponding to a truncation point applied to *p* proportion of survival. Significance of the heritability estimates was tested using z-score against a large random normal distribution [29].

## Results

### Population structure and characteristics of the data

A total of 235,060 individual larvae were analysed from offspring of 18 sires and 30 dams recorded over a nine-year period from 2006 to 2014 (Table 1). In the first two years (2006 and 2007), offspring from only one female was reared for this experiment. In subsequent seasons, the number of females that successfully spawned increased, averaging 3–4 females per breeding season (year). However, some females and males were repeatedly used in 2008–2014. Hence, the unique number of males and females in this study was 6 and 10, respectively.

**Table 1:** Number of sires, dams and offspring and survival rate (%) from hatching to puerulus

Year	Sire	Dam	Offspring	Survival (%)^a^
2006	1	1	14,800	1.37
2007	2	2	24,000	3.19
2008	2	3	31,600	n.a.
2009	2	4	30,000	19.17
2010	2	4	35,000	34.80
2011	3	4	13,200	14.32
2012	2	5	36,360	21.60
2013	2	4	17,100	8.55
2014	2	3	33,000	18.00
All years	18	30	235,060	17.67

A total of 30 families in which there were 20 half-sib groups (some males were mated with multiple females). Total number of unique males and females = 6 and 10, respectively (the same males and females were repeatedly paired in years 2008–2014)

^a^Mean survival after metamorphosis (calculated as percent difference from stocking), n.a. = not available

**Fig. 1:**
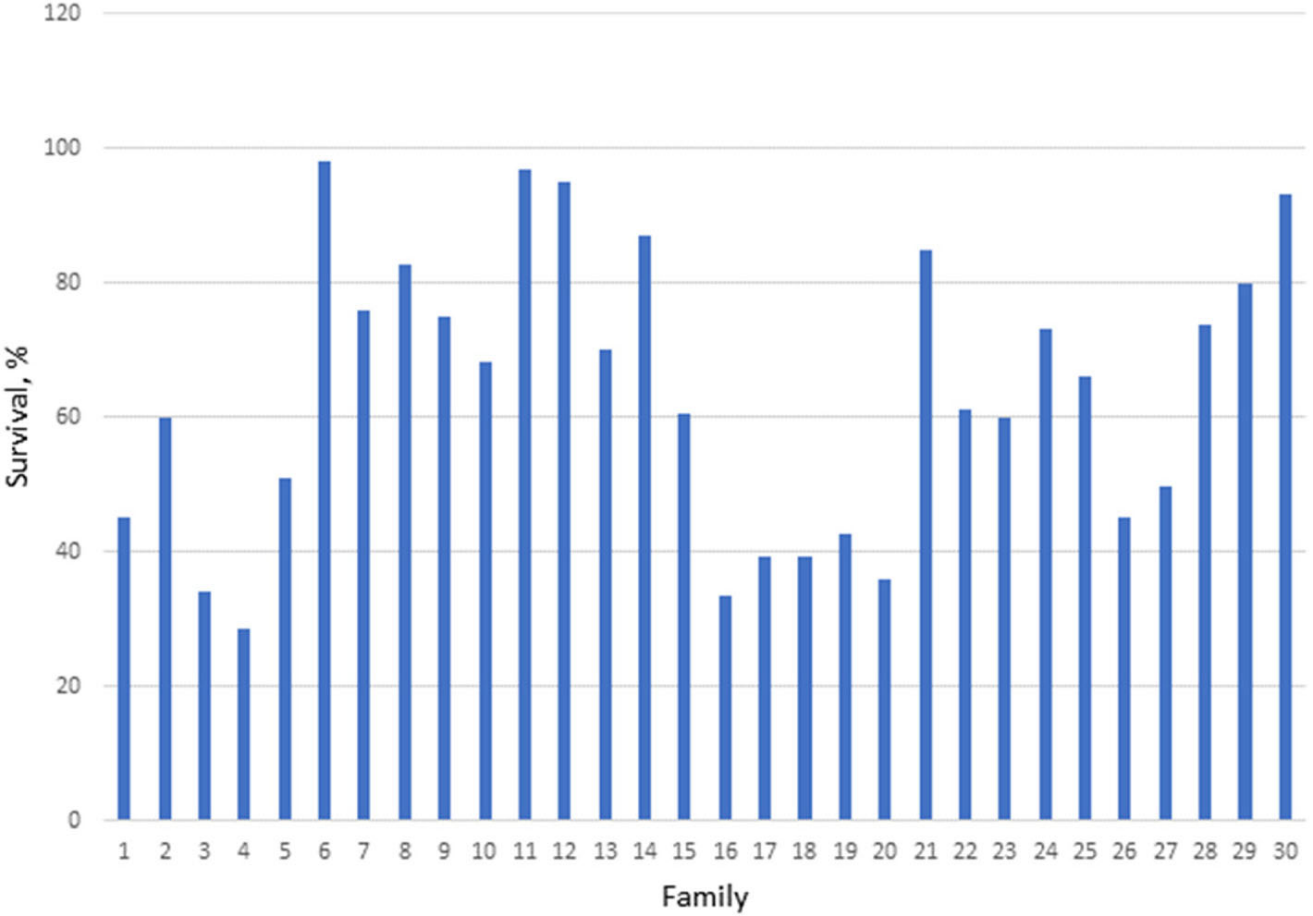
Variation in survival rate (%) from hatching to instars 1–6 (S1) among breeding pairs (family) in the population

Average survival rates of *S. verreauxi* during the five stages of rearing show significant variation (*P* < 0.05) among full-sib families or breeding pairs recorded over the nine-year period (Fig. 1 and Additional file 2: Figure S1). The variation within full-sib families was also significant (*P* < 0.05). Taking survival from hatching to instars 1–6 (S1) as an example, the difference among the families that had the highest and lowest survival was 69.4% (Fig. 1). These differences for S2, S3, S4 and S5 were 92.3, 66.4, 98.5 and 79.4%, respectively (Additional file 2: Figure S1). In a similar manner, there were also substantial differences in the coefficient of variation (CV) for survival traits among families. For example, the CV for S1 ranged from 14 to 158%.

The mean survival rates of the whole population averaged over nine years is given in Table 2. The survival rates in the early rearing stages (S1, S2 and S3) were higher than those in later periods (S4 and S5). Analysis of variance using threshold generalised model approach showed significant differences in survival rates among the five stages of early rearing in *S. verreauxi*, being lowest (59.5%) for S4 and highest (about 74.8%) for S2 (*P* < 0.01).

**Table 2:** Survival rates at specific stages of rearing (S1, S2, S3, S4 and S5), averaged over nine years

Trait	Unit	*n*	Mean Survival	SD	Min	Max
S1	%	235,060	64.6	20.6	28.6	98.0
S2	%	144,848	76.6	25.4	7.2	99.5
S3	%	115,193	64.1	19.7	29.1	95.5
S4	%	71,456	59.5	19.8	11.0	98.6
S5	%	8429	55.4	27.2	0.0	79.4

Min = lowest survival family and max = highest survival family

S1 = Early-phyllosoma stages (instars 1–6), S2 = Mid-phyllosoma stages (instars 7–12), S3 = Late-phyllosoma stages (instars 13–17), S4 = Metamorphosis and S5 = Puerulus stage

Offspring survivals of three replicate parental pairs (breeding cohorts) that were used repeatedly in years 2009–2014 were similar during the early phase of rearing (S1 and S2). However, there were significant differences (*P* < 0.05) in S3, S4 and S5 among the three cohorts (Additional file 3: Figure S2). The CV range for metamorphosis survival among these cohorts was 27–60%.

### Non-genetic effects on survival over years

General linear model was also used to test the effect of supplementary feeds (Artemia, mussel gonad and formulated diet) on survival. Supplementing live feeds with formulated diet did not have significant effects on survival rates (*P* > 0.05).

**Fig. 2:**
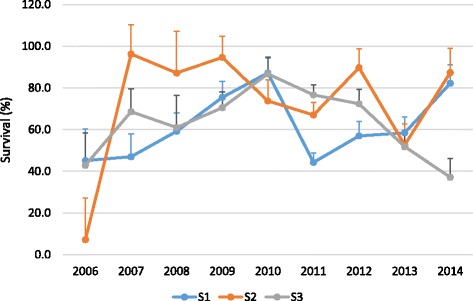
Survival rates during the early stages of larval rearing: S1, S2 and S3 for all offspring over nine years of study (significant differences among years at *P* < 0.001) S1 = Early-phyllosoma stages (instars 1–6), S2 = Mid-phyllosoma stages (instars; 7–12) and S3 = Late-phyllosoma stages (instars 13–17)

On the other hand, survival rates (S1 – S5) differed statistically between spawning seasons (or spawning years). Yearly survival rates for S1, S2 and S3 showed an increasing trend over the nine years of the study (Fig. 2). For a given measure of survival such as during metamorphosis (S4), the differences among the nine years studied were significant at 0.1% (*P* < 0.001). Within a spawning year (season), the survival rates at different rearing stages also differed statistically (*P* < 0.05). A linear regression showed an annual improvement in metamorphosis survival by about 2.86% (*R*^2^ = 0.07).

### Heritability and common full-sib effects

Heritability (h^2^) for the five measures of early survival in *S. verreauxi* were estimated using two different statistical models: i) Linear General Mixed Model (LMM, model 1), ii) Threshold Logistic Generalised Linear Mixed Model (TLGM, model 2).

The heritability obtained from model 1 (LMM) for the survival traits before metamorphosis (S1, S2 and S3) were moderate (Table 3). The h^2^ estimates generally varied with rearing phases, i.e. higher in the early than later phase of rearing (h^2^ = 0.15 for S3 vs. 0.03 for S4). The heritability for S5 were not different from zero, due to the limited number of families available during this rearing phase.

**Table 3:** Heritability (± standard errors) for five measures of survival during the early phase of rearing

Traits	Model 1			Model 2	
	h^2^	c^2^		h^2^	c^2^
S1^a^	0.47 ± 0.02	0.03 ± 0.02		0.50 ± 0.01	0.03 ± 0.03
S2^a^	0.23 ± 0.07	0.04 ± 0.02		0.33 ± 0.07	0.04 ± 0.02
S3	0.15 ± 0.04	0.10 ± 0.07		0.05 ± 0.01	0.18 ± 0.08
S4	0.03 ± 0.01	0.12 ± 0.06		0.05 ± 0.01	0.12 ± 0.06
S5^a^	0.02 ± 0.06	0.04 ± 0.01		0.02 ± 0.08	0.01 ± 0.02

Model 1 = Linear Mixed Model, Model 2 = Generalised Linear Mixed Model using a logit link function

^a^Sire and Dam Model [15] was used because both model 1 and 2 were not converged

S1 = Early-phyllosoma stages (instars 1–6), S2 = Mid-phyllosoma stages (instars; 7–12), S3 = Late-phyllosoma stages (instars 13–17), S4 = Metamorphosis and S5 = Puerulus stage

Due to binary characteristics of survival measures, the heritability’s were also estimated from the model 2. The h^2^ estimates from model 2 were low to moderate (ranging from 0.01 to 0.50). Across the models, the estimates of heritability for survival at five different time points during the early phases of rearing were significant (*p* < 0.05), indicating that there are additive genetic variations in these characters to be improved via selection.

The common full-sib effects (c^2^) accounted for 3 to 18% of total variance for survival traits. The c^2^ estimates were generally similar between the two statistical models.

### Phenotypic and genetic correlations

Phenotypic and genetic (r_g_) correlations among the five measures of early survival were moderate to high and all positive (Table 4). With only two exceptions, all the phenotypic and genetic correlations were significant. The magnitude of the genetic correlations between measures of survival in successive rearing periods were greater than those that are further apart, such as r_g_ = 0.712 between S1 and S2 vs. 0.21 between S1 and S5. The estimates of phenotypic correlations of the survival traits had similar sign and magnitude as those obtained for the genetic correlations.

**Table 4:** Phenotypic (above) and genetic (below) correlations among survival traits

Traits	S1	S2	S3	S4	S5
S1		0.80 ± 0.05	0.68 ± 0.08	0.42 ± 0.08	0.73 ± 0.25
S2	0.88 ± 0.10		0.69 ± 0.03	0.45 ± 0.03	0.58 ± 0.92
S3	0.54 ± 0.31	0.89 ± 0.09		0.66 ± 0.02	n.e.
S4	0.78 ± 0.18	0.85 ± 0.13	0.67 ± 0.05		n.e.
S5	n.e.	0.22 ± 1.06	n.e.	n.e.	

S1 = Early-phyllosoma stages (instars 1–6) survival, S2 = mid-phyllosoma stages (instars; 7–12), S3 = late-phyllosoma stages (instars 13–17), S4 = Metamorphosis survival and S5 = Puerulus stage

n.e. = not estimable due to non-convergence of the log likelihood

## Discussion

### Larval survival is heritable and should respond to selection

Our study reports, for the first time, a set of genetic parameters (heritability and correlations) for a spiny lobster species, *S. verreauxi*. Difficulties in the larval culture of *S. verreauxi* has previously precluded analysis of genetic parameters in lobster species. The successful breeding and closure of the production cycle for *S. verreauxi* enabled the systematic recording of family data over a nine year period to estimate heritability and correlations for larval survival traits at different stages in culture. Both linear mixed and generalised threshold models showed the additive nature of genetic components for five measures of larval survival during hatchery production. So the progeny from high breeding value females (e.g. animal ID C85) performed better in culture both within and between years. This was particularly true for mean survival at metamorphosis. Successfully culturing phyllosoma through metamorphosis has been a major bottleneck in production of all spiny lobster species [1]. Our results indicate that in addition to the improvement of exogenous factors controlling variation in survival metamorphosis [23], there are prospects for further genetic improvement of survival through selective breeding to increase production of *S. verreauxi* seed.

To date, only a few studies have reported heritabilities for early rearing survival for farmed aquaculture species and these mainly involved salmonids [30, 31] and tilapia [19]. The literature estimates to date have been reported mostly for survival during grow-out stage, i.e., from physical tagging when post-larvae or fry reach a suitable size reared until final harvest. The additive genetic components also exist for survival during the grow-out in commonly farmed species such as tilapia [32], carps [16] or marine shrimps [33]. The estimates of heritability for on-growing survival were reported repeatedly in several studies to be low, with a few exceptions of the moderate magnitude [15]. The heritability for larval survival may be population-dependent and the survivals at different stages of early growth may be under different genetic control [19]. Also note that there is no published information regarding the maternal and common environmental effects (c^2^) on survival traits during the early phase of rearing in crustacean species. The c^2^ estimates in our study ranged from 3 to 18%.

### Genetic correlations

Our results also show strong genetic associations among the five measures of larval survival. Of practical significance is the close to perfect (unity) genetic correlations between S2 (instars 7–12 survival) and S4 (metamorphosis survival), suggesting that selection for larval survival post instar7 can improve the number of larvae during metamorphosis (S4). The increased survival rate during this period is crucial to commercial hatcheries to enhance revenue and return. However, selection for increased early survival (S1 or S2) may not capture all genetic expressions in subsequent rearing periods, as indicated by the genetic correlation estimates that were significantly different from one, such as between S2 and Puerulus survival. In a selection program for rainbow trout over 10 generations, Vehviläinen et al. [18] also reported a positively moderate (0.3 ± 0.16) genetic correlation between survivals at early and late fingerling stages. Thoa et al. [19] also found high and positive genetic correlations between survival from hatching to 20 d and from 21 to 62 d (when the animals were physically tagged). Our results are broadly in agreement with these studies on rainbow trout *O. mykiss* and Nile tilapia *O. niloticus*, suggesting that the survival rates at different rearing periods may be under alternative genetic control and that they should be treated as genetically different traits in selective breeding programs. However, further studies are needed to have a better understanding about genetic architecture of age-specific survival trait across life stages in lobster species.

### Environmental contributions

Despite the significant heritability in all the statistical models used, the estimates decreased as the larvae developed. This is as expected because the larval survivals during the early phases of growth development are strongly influenced by intrinsic environmental factors, e.g. Thoa et al. [17]. A number of studies have shown that lobster larval survival depends strongly on various rearing conditions and management practices, such as water quality parameters [3, 34], light regimes [23], background of rearing facility and stocking density [35], supplementary feeds and diets [36, 37]. In the present study, attempts were made to provide a homogeneous rearing environments and husbandry practices, but variation in the rearing tanks and hatchery practices between years would have affected survivals. Information on particular rearing tanks, when available, was included in our statistical models and when combined with spawning year as a contemporary group tank effect was found to be not significant. The effects of feed items on larval survival was also non-significant (*P* > 0.05). Studies in fish and other species of spiny lobsters [38] also suggest that increasing female body weight was associated with larger larval body size and subsequently improving survival rates in the early phase of rearing [19]. Taken collectively, our results together with those reported in the literature suggest that in breeding programs and commercial production hatcheries, survival rates can be improved by minimising environmental impacts and improving farming practices, as reported in fish species [39].

Although basic information about quantitative genetic inheritance is reported for survival traits in spiny lobsters, the design of this study had been adjusted to fit biology of the species as well as the availability of facilities and resources at our station. Genetic parameters obtained here are likely somewhat biased due to possible confounding of environmental and genetic effects. Hence, when production cycle is fully controlled for *S. verreauxi*, breeding a larger number of families using new broodstocks should be made to frequently update heritability and genetic correlations for survival traits especially those from hatching to settlement. Furthermore, upgrading the hatchery capacity should be conducted to increase availability of spawning and rearing tanks to accommodate a larger number of families per year (e.g., 5–10 families per year). This would enable larval rearing of families in replicate tanks to minimise possible environmental differences in survival rates among families and the family (or genotype) by environment effects [40, 41], as well to enable a separation of the additive genetics from tank effects [42]. A routine data collection in larger pedigrees would allow the estimation of genetic parameters with higher accuracy which are necessary to guide future breeding programs in lobsters species.

## Conclusions

The new set of genetic parameters obtained for *S. verreauxi* assisted our understanding of the genetic architecture of quantitative traits of commercial importance. This information will aid in the design of future breeding program for lobster species. Our estimates of heritability and genetic correlations among five measures of larval survival indicated that it is possible to improve hatchery production of this species, especially during the key bottle-neck around metamorphosis, and that selection for increased survival in the early phase can improve survival rates in subsequent rearing periods. Successful captive breeding of *S. verreauxi* and improving seed quality through genetic selection are likely to help facilitate commercial production of this sector.

## Additional files

Additional file 1: **Table S1.** Characterization of microsatellite loci (DOCX 16 kb)

Additional file 2: **Figure S1.** Variation in survival rates among families (DOCX 33 kb)

Additional file 3: **Figure S2.** Variation in survival rates among breeding cohorts (DOCX 32 kb)

## Abbreviations

S1: Early-phyllosoma stages (instars 1–6)
S2: Mid-phyllosoma stages (instars; 7–12)
S3: Late-phyllosoma stages (instars 13–17)
S4: Metamorphosis
S5: Puerulus stage
