# Supplementary material for: Can metamorphosis survival during larval development in spiny lobster Sagmariasus verreauxi be improved through quantitative genetic inheritance?
Source: BMC Genet. 2018 May 4;19:27. doi: 10.1186/s12863-018-0621-z (PMC5936031; doi:10.1186/s12863-018-0621-z)
Supplement: Supplementary file 1 — Table S1. Characterization of microsatellite loci (DOCX 16 kb) [file 12863_2018_621_MOESM1_ESM.docx]

**Table S1** (update the table later): Characterization of microsatellite loci isolated from 166 individuals of *Sagmariasus verreauxi* collected from Tasmania, Australia. *PIC*, polymorphic information content; *N,* number of individuals successfully amplified; *N_A_*, number of alleles; *H_O_*, observed heterozygosity; *H_E_*, expected heterozygosity; *F_IS_*, inbreeding coefficient; * significant departure from HWE.

| Locus | Repeat motif | Primer sequences  (5’ – 3’) | Size range (bp) | PIC | N | N_A_ | H_0_ | H_E_ | F(Null) |
| --- | --- | --- | --- | --- | --- | --- | --- | --- | --- |
| **SV08** | (AC)_7_ | F: CCGAGTACACGTCTCCCAAC  R: CCTCCGCCTTCCATACATTA | 160-162 | 0.268 | 299 | 2 | 0.28 | 0.32 | 0.07 |
| **SV10*** | (AG)_7_ | F: ATGGTGATGACGGGTGACAT  R: TCATCAACACCACTCCCTCC | 147-157 | 0.389 | 299 | 3 | 0.35 | 0.51 | 0.18 |
| **SV15** | (AC)_8_ | F: ACCATGAGGAGGCAAACTGA  R: CCTGAAGACCAAGCTGGATG | 147-149 | 0.374 | 299 | 2 | 0.47 | 0.50 | 0.03 |
| **SV16** | (AT)_9_ | F: CGACCCTGACATCTTCCTTG  R: GCATATACCATGGGCGAGAT | 197-205 | 0.507 | 299 | 4 | 0.53 | 0.59 | 0.05 |
| **SV19** | (ACC)_7_ | F: CTTGGGAACACGGCTGTAAC  R: CCCAAACTCCACCCTATTCC | 167-170 | 0.068 | 299 | 2 | 0.06 | 0.07 | 0.08 |
| **SV33** | (AT)_9_ | F: CAATGCGCACATCCATAGAA  R: ACCTCCCAAGCACAGAAACC | 264-273 | 0.569 | 299 | 5 | 0.60 | 0.62 | 0.01 |
| **SV60*** | (AG)_6_ | F: ATCTGCCTCCCAGGAACAAT  R: ACTGAGGCAGGGACAAATGA | 140-159 | 0.711 | 299 | 6 | 0.74 | 0.75 | -0.01 |
| **SV63** | (AT)_7_ | F: TTCACTTCTTGTTCACAGCCC  R: AGTTGGCTCGCAATCCATTA | 160-166 | 0.538 | 299 | 3 | 0.61 | 0.62 | 0.01 |
| **SV67*** | (AG)_6_ | F: AGCATCAGGAAGGAGTGCTG  R: TTCTTCCGTTGCCTCATTGT | 193-206 | 0.529 | 299 | 4 | 0.68 | 0.59 | -0.07 |
| **SV75*** | (AC)_7_ | F: ACCTTGCGATTCGTTCGTTA  R: GGAAACACTGTCTTGCCCAC | 210-218 | 0.583 | 299 | 4 | 0.72 | 0.65 | -0.06 |
| **SV78*** | (AGG)_6_ | F: CGGTGTGAAGGTGAGGTGA  R: CCTGCTGTATGTCTGCACCA | 187-203 | 0.650 | 299 | 4 | 0.89 | 0.71 | -0.12 |
