# Supplementary material for: Can metamorphosis survival during larval development in spiny lobster Sagmariasus verreauxi be improved through quantitative genetic inheritance?
Source: BMC Genet. 2018 May 4;19:27. doi: 10.1186/s12863-018-0621-z (PMC5936031; doi:10.1186/s12863-018-0621-z)
Supplement: Supplementary file 2 — Figure S1. Variation in survival rates among families (DOCX 33 kb) [file 12863_2018_621_MOESM2_ESM.docx]

**Figure S1**

| 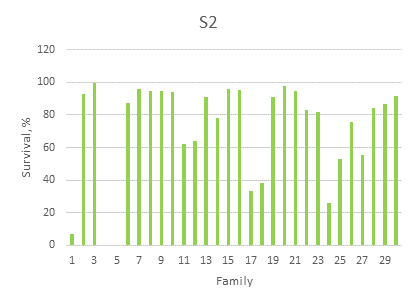 | 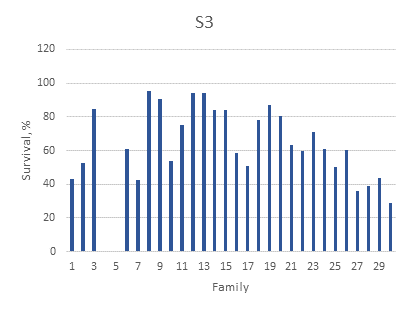 |
| --- | --- |
| 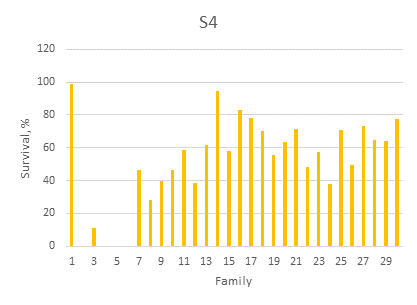 | 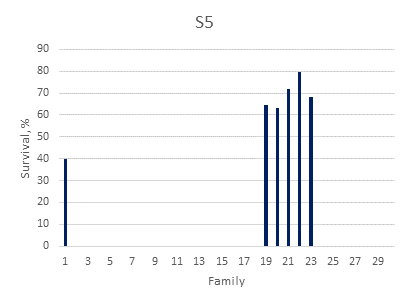 |
| Figure S1: Variation in survival rate (%) from instar7-12 (S2), instar 13-17 (S3), metamorphosis (S4) and puerulus (S5) among breeding pairs (family) in the population, P < 0.001 | |
