# Supplementary material for: Can metamorphosis survival during larval development in spiny lobster Sagmariasus verreauxi be improved through quantitative genetic inheritance?
Source: BMC Genet. 2018 May 4;19:27. doi: 10.1186/s12863-018-0621-z (PMC5936031; doi:10.1186/s12863-018-0621-z)
Supplement: Supplementary file 3 — Figure S2. Variation in survival rates among breeding cohorts (DOCX 32 kb) [file 12863_2018_621_MOESM3_ESM.docx]

**Figure S2**

| 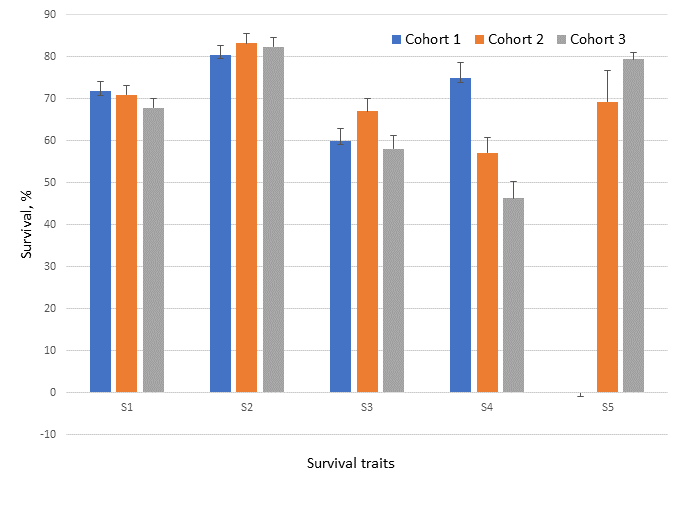 |
| --- |
| Figure S2: Variation in survival rate (%) from hatching to instars 1-6 (S1), instar7-12 (S2), instar 13-17 (S3), metamorphosis (S4) and puerulus (S5) among three common cohorts used repeatedly over years 2009-2014 (P < 0.001) |
